# Safety Planning: Why It Is Essential on the Day of Discharge From In-patient Psychiatric Hospitalization in Reducing Future Risks of Suicide

**DOI:** 10.7759/cureus.20648

**Published:** 2021-12-23

**Authors:** Haley Schuster, Nathan Jones, Syed F Qadri

**Affiliations:** 1 Psychiatry, Creighton University School of Medicine, Omaha, USA; 2 College of Allied Health Professionals, University of Nebraska Medical Center, Omaha, USA

**Keywords:** psychiatric hospitalization, post-discharge, mental illness, suicide prevention, safety planning intervention

## Abstract

Individuals who suffer from mental illness are at an increased risk for suicide. That risk is substantially higher in the post-discharge period from psychiatric hospitalization. Safety planning intervention (SPI) is a common intervention tool that is utilized to mitigate the risk of suicide. Current research notes promising results of SPI use in the emergency department (ED); however, there is limited research regarding SPI use during psychiatric hospitalization on the day of discharge. This paper aims to evaluate current research on the topic and establish a need for more widespread use of SPI during psychiatric hospitalization.

## Introduction

According to the National Institute of Mental Health, about one in five adults in the USA live with a mental illness. In 2019, 51.5 million people suffered from mental illnesses with varying degrees of severity. Many safety concerns arise from patients suffering from psychiatric illness [1]. Education about mental health illnesses and understanding the specific safety concerns that may arise from those illnesses might aid in mitigating negative consequences. Adverse complications that arise from mental health illness include, but are not limited to, family conflict, social segregation, work conflict, educational problem, financial trouble, legal problem, substance misuse, self-harm, and suicide [2].

Patients diagnosed with a psychiatric illness are at a heightened risk of suicide. Suicide is the 10th leading cause of death in the USA. In people aged 10-34, suicide is the second leading cause of death, and for those aged 35-44, it is fourth [3]. Understanding an individual’s suicide risk is a very important clinical feature that clinicians must address during mental health treatment [4]. The safety and physical well-being of patients remains a top priority for healthcare professionals.

Suicide risks vary between individuals as a consequence of mental illness diagnosis, age, and sex. Patients diagnosed with schizophrenia, most commonly diagnosed in patients 35 years and younger, have the highest rate of suicide, with a lifetime risk of 4.9% [4,5]. In patients diagnosed with recurrent depression, suicide rates were the highest for patients 60 years old and older. For patients aged 36-60, suicide rates were the highest in males diagnosed with alcohol use disorder and females diagnosed with personality disorders. The prevalence of suicide is relatively low in people suffering from serious mental illness. The risk, however, is significantly increased in people who are hospitalized for treatment for their psychiatric illness. The prevalence of suicide in patients who required hospitalization was found to be 37% for males and 57% for females [4]. Suicide rates were the highest at post-psychiatric hospitalization, especially at the three-month mark, and the risk remains elevated even years later. Given the individual variability of suicide risk, individualized safety planning is a vital tool of healthcare providers in suicide risk mitigation [6].

## Materials and methods

Lasting Hope Recovery Center is an adult in-patient psychiatric facility located in Omaha, NE, USA, that provides acute and subacute care. Dr. Syed F. Qadri, an attending physician and psychiatrist at Lasting Hope Recovery Center, has developed and implemented his own step-by-step process to assess for and mitigate the risk of harm prior to discharge from psychiatric hospitalization (Table 1).

**Table 1:** Dr. Syed F. Qadri’s Steps of Safety Planning

Steps	Assessed
1	The presence of suicidal ideation (SI), homicidal ideation (HI), auditory hallucinations (AH), visual hallucinations (VH), depressive symptoms, paranoia, and delusional thinking
2a	The patient’s plan/goals immediately following discharge
2b	The patient’s future plans regarding education, occupation, and interpersonal relationships
3a	What steps the patient would take in the event of safety concerns (i.e., SI, HI, etc.)
3b	If Step 3a coping skills are not effective, then call 911. Educate the patient that law enforcement officers are trained to recognize this type of distress and to take them to the nearest hospital for evaluation
3c	Make the following recommendations: ask the patient their willingness to continue medications as prescribed, attend psychiatric outpatient appointments, and abstain from illicit drugs and alcohol
3d	The patient will be provided discharge instructions/safety plan worksheet

Step 1 of this safety plan assesses for notable risk factors of suicide. Step 2 permits providers to understand to what extent the patient is future-oriented. It also implies the degree of their hopelessness. Step 2a looks at immediate future orientation, whereas Step 2b looks at remote future orientation. Step 3 serves the purpose of mitigating the risk of future harm [7]. The safety plan worksheet mentioned in Step 3d is shown in Table 2 and Table 3. This worksheet is a form that the patients are asked to work on in their downtime. Once it is completed, the worksheet is put into the patient’s chart on EPIC prior to discharge.

The safety plan worksheet shown in Table 2 and Table 3 is the product of an action plan that was developed several years ago when Catholic Health Initiatives (CHI), the health organization to which Lasting Hope Recovery Center belongs, was seeing an increased rate in suicides. At the time, CHI did not have a formal safety plan that could be shared with schools (for the child and adolescent patients), outpatient providers, loved ones, or when the patient returns to the ED. The literature search done at the time did not provide an evidence-based tool, so a team of therapists and nurses designed the worksheet shown below. This safety plan worksheet is currently implemented in all adult inpatient, child and adolescent, and geriatric psychiatry units in CHI. It is also part of the after-visit summary (AVS) so that patients, parents, caregivers, or legal guardians receive a copy of the safety plan worksheet that the patient filled out on discharge. The AVS is a part of the continuum of care notification handoffs to schools and providers outside the CHI system. Anecdotally, this safety plan worksheet has been successful at reducing suicide rates, reducing the number of psychiatric hospitalizations, and increasing the time between psychiatric hospitalizations. To our knowledge, no formal research has been conducted to evaluate the efficacy of this safety plan worksheet.

**Table 2:** Safety Plan Worksheet Provided to Patients Upon Discharge

Safety Plan Worksheet	
These are my warning signs that a crisis may be developing:	
Thoughts:	
Feelings:	
Behaviors:	
Symptoms:	
I find that the following people, places, and situations are stressors for me:	
1.	
2.	
3.	
My most effective coping strategies:	
These coping strategies help lessen my distress: (please circle all those that apply and feel free to add more):	Listen to music, read a book, wrap in a blanket, talk to a trusted adult, talk to a trusted friend, call a family member, spend time in the comfort room, count to 10, play with a pet, guided imagery, spend time in a dim-lit room, read a spiritual book or reflection, read inspirational quotes, write a letter, do artwork, draw a picture, positive self-talk, think a positive thought about self, take space, relaxation, journal about feelings
Safe soothers (fill in the blank)	Sensory soother __________________________________________________________________________________________________________________________________________________________________________ Visual soother ____________________________________________________________________________________________________________________________________________________________________________ Scent soother _____________________________________________________________________________________________________________________________________________________________________________ Touch soother _____________________________________________________________________________________________________________________________________________________________________________ Hearing soother ___________________________________________________________________________________________________________________________________________________________________________ Taste soother ____________________________________________________________________________________________________________________________________________________________________________

**Table 3:** Safety Plan Worksheet Provided to Patients Upon Discharge (Continued)

Safety Plan Worksheet	
These are healthy activities I can use to distract myself (please circle those that apply):	Go for a walk, exercise, drink water, take prescribed medication, biofeedback, deep breathing, healthy snack
I know my coping strategies are working when I:	
When I need to reach out to someone, I will contact these social supports:	
Friends:	
Family:	
Others:	
Professionals I can contact in a crisis (list name and phone #):	
Psychiatric provider:	
Therapist:	
Friends:	
Other:	
Crisis line, local:	
Crisis line, national:	National Hotline: 1-800-273-TALK (8255) NIMH/NAMI Hotline
CHI health Omaha crisis lines and supports: 402-717-HOPE (4673) Safe Harbor: 402-715-4226 AA Omaha central office: 402-556-1880 Boys Town hotline: 1-800-448-3000 Nebraska family helpline: 1-888-866-8660	
In order to keep myself safe, I will remove or safely store items that might be harmful to me: DROP DOWN	
Firearms:	
Medications:	
Household poisons:	
Sharps or other dangerous objects:	
If I still feel unsafe, I will go to the nearest hospital or emergency department (ED), or call 911:	
What might prevent me from following this Safety Plan?	
Who will I share this plan with?	
Where will I keep this plan?	

The safety plan worksheet provided to patients in the CHI system prior to discharge from psychiatric hospitalization has many similarities to the Brown and Stanley model, which implements six key steps to mitigate the risk of suicide. In the Brown and Stanley model, Step 1 asks the patient to list warning signs that suggest they are heading toward an emotional crisis [7], and Step 2 asks the patient to list internal coping strategies [7]. In Step 3, the patient is asked to list social contracts that may distract the patient from the crisis [7], whereas Step 4 asks the patient to list family members or friends who may offer help [7]. Step 5 asks the patient to list professionals and agencies to contact for help [7], and Step 6 looks into making the patient’s environment safe by removing firearms [7]. Patients are also assessed on their likelihood to utilize each step in this model [7]. The answers the patient provided to each of these steps create an individualized safety plan, “a prioritized list of coping strategies and sources of support patients can use who have been deemed to be at high risk for suicide” [7]. The goal of the safety plan worksheet is to help the patient create a safety plan that is “brief, in the patient’s own words, and is easy to read” [7].

## Results

Safety planning or safety planning intervention (SPI) has largely replaced the once-popular no-suicide contract. A no-suicide contract is a verbal or written agreement between a patient and their physician in which the patient contractually agreed to abstain from suicidal behavior and seek professional help in times of crisis [8]. Although no-suicide contracts may ease the clinician’s concern regarding potential suicide risk, there is no evidence to support the effectiveness of no-suicide contracts for reducing suicidal behavior [8-11].

In addition to the absence of evidence concerning the effectiveness of no-suicide contracts, these contracts may actually pose a risk. “A unilateral or authoritarian style of implementing contracts may cause patients to feel threatened or coerced” [12] and may threaten the therapeutic alliance of the patient and physician. As a consequence, “patients may withhold information about their desire to kill themselves for fear that they will disappoint their treating clinicians by violating the contract” [8]. Clinical guidelines caution against utilizing no-suicide contracts to mitigate the risk of suicide because they may make it more difficult to determine the patients’ actual suicidal risk [8,11,13].

A collaborative approach to safety planning has been shown to be a more effective means of mitigating suicide risk [14]. This approach is designed “to foster a stronger therapeutic alliance and increase motivation within a suicidal patient” [14]. The safety plan itself comprised six standard components: (a) recognizing warning signs of an impending suicidal crisis, (b) employing internal coping strategies, (c) utilizing social contacts as a means of distraction from suicidal thoughts, (d) contacting family members or friends who may help resolve the crisis, (e) contacting mental health professionals or agencies, and (f) reducing the potential use of lethal means [7,8,14]. It is important that the safety plan “is brief, is in the patient’s own words, and is easy to read” [7]. By involving the patient in the process of creating a safety plan, the safety plan created is unique to each patient. In addition, including the patient in this process may increase motivation for the patient to utilize their safety plan [8].

Research suggests that utilizing SPI in the emergency department (ED) is superior to “usual care” [15]. Usual care is defined as an initial assessment by an ED nurse or social worker, followed by a secondary evaluation by an ED physician [15]. One study demonstrated that SPI “was associated with 45% fewer suicidal behaviors in the six-month period following the ED visit compared with usual care” [15]. In addition, patients who received SPI in the ED were more than twice as likely to attend at least one outpatient mental health visit [15]. This statistic alone is perhaps an argument in and of itself to support the more widespread use of SPI.

Up to 80% of patients who attempt suicide do not receive treatment in an outpatient clinic following their initial assessment in the ED [16-20]. The level of lethality of the self-harm attempt has been shown not to be significantly related to the likelihood of receiving follow-up care [18]. Olfson et al. found that less than half “of deliberate self-harm treatment episodes that involved a highly lethal method of injury (use of firearms, drowning, suffocation, falling, use of fire, or use of motor vehicles to inflict self-injury) included outpatient mental healthcare in the following 30 days” [18]. Of patients who attempted suicide and received follow-up care, up to 38% terminated care within the first three months following their suicide attempt [16,20,21]. This statistic is particularly worrisome because “the first three months following a suicide attempt is when individuals are at the highest risk of additional suicidal behavior [21].”

Patients are at an increased risk of suicide within the first few months following post-discharge from a psychiatric hospitalization [6,22-28]. One meta-analysis found that “the post-discharge suicide rate was approximately 100 times the global suicide rate during the first three months after discharge from a psychiatric hospitalization [6].” Even after many years, patients who were previously hospitalized for psychiatric conditions have suicide rates that are approximately 30 times higher than typical global rates [6]. Those patients especially at high risk for suicide in the post-discharge period have a history of self-harm or suicide attempt and endorse depressive symptoms and hopelessness [6,23,25,27-30].

However, there is limited research on safety planning in the post-discharge period from psychiatric hospitalization. Leonard et al. found that 96% of patients had a copy of their safety plan one week post-discharge [31]. However, that same study found that only 36.8% of patients had used their safety plan since discharge and that only 67.9% of patients found SPI to be helpful [31]. More research is needed to evaluate and maximize the effectiveness of safety planning in the post-discharge period following psychiatric hospitalization.

## Discussion

Veteran Affairs (VA) was one of the first entities to incorporate the widespread use of SPI on the day of discharge from psychiatric hospitalization. As early as 2013, the VA/DoD Clinical Practice Guideline for Assessment and Management of Patients at Risk for Suicide included “safety planning that is developed collaboratively with the patient should be part of discharge planning for all patients who were evaluated with high acute risk for suicide before being released to a lower level of care” in their recommendations [32]. The safety planning described in the VA/DoD Clinical Practice Guideline for Assessment and Management of Patients at Risk for Suicide closely resembled the Brown and Stanley model [7]. Despite the limited data on suicide prevention at the time, the VA recognized the importance of implementing safety plans that were individualized for the patient and that were created through a collaborative effort between the provider and the patient [7,32].

Research examining the safety planning that is utilized at the VA suggests efficacy in this approach. Kayman et al. reason that having a physical copy of the safety plan that is easily accessible to the patient “reduces the burden of problem-solving when a crisis is looming and the ability to think clearly is impaired” [33]. The details of an individualized safety plan serve as “potent reminders” [33] that the patient is “neither helpless nor alone” [33]. This is supported by a 2015 study that looked at the attitudes suicidal veterans had toward safety planning. In this study, some veterans reported a reduction in their symptoms as a result of using the coping strategies listed in their safety plan [34]. Others found that simply looking at the list of coping strategies documented in their safety plan had a calming effect [34]. In addition, there were some veterans who shared their safety plans with friends in family members “not only to inform them of its content but also to enlist their support in applying the strategies and using the resources listed” [34].

As new data is emerging, it seems to support what was postulated in the studies that assessed SPI use in veterans. Two meta-analyses found that suicide prevention interventions, safety planning being the most utilized [35], reduce suicidal behavior [35,36]. In addition, Doupnik et al. found that the use of suicide prevention interventions increases the likelihood of patients engaging in follow-up mental healthcare [36]. Although current research is promising, continued research is needed to further assess the effectiveness of safety planning in the post-discharge period following psychiatric hospitalization.

## Conclusions

This paper reviewed current research on safety planning intervention (SPI) while also establishing a need for future research. The heightened risk of suicide in the post-discharge period from psychiatric hospitalization has been well documented. Current research suggests that SPI is effective at mitigating suicidal behavior when utilized in the ED. However, there is limited research regarding SPI use in the post-discharge period from psychiatric hospitalization. Given the promising data regarding SPI use in the ED, we recommend that future research explore SPI and safety planning effectiveness on the day of discharge from psychiatric hospitalization.
